# DDQN with Prioritized Experience Replay-Based Optimized Geographical Routing Protocol of Considering Link Stability and Energy Prediction for UANET

**DOI:** 10.3390/s22135020

**Published:** 2022-07-03

**Authors:** Yanan Zhang, Hongbing Qiu

**Affiliations:** School of Information and Communications, Guilin University of Electronic Technology, Guilin 541004, China; qiuhb@guet.edu.cn

**Keywords:** unmanned aerial vehicles (UAVs), routing protocol, deep double Q network (DDQN), prioritized experience replay, link stability, autoregressive integrated moving average (ARIMA), energy prediction

## Abstract

Unmanned aerial vehicles (UAVs) are important equipment for efficiently executing search and rescue missions in disaster or air-crash scenarios. Each node can communicate with the others by a routing protocol in UAV ad hoc networks (UANETs). However, UAV routing protocols are faced with the challenges of high mobility and limited node energy, which hugely lead to unstable link and sparse network topology due to premature node death. Eventually, this severely affects network performance. In order to solve these problems, we proposed the deep-reinforcement-learning-based geographical routing protocol of considering link stability and energy prediction (DSEGR) for UANETs. First of all, we came up with the link stability evaluation indicator and utilized the autoregressive integrated moving average (ARIMA) model to predict the residual energy of neighbor nodes. Then, the packet forward process was modeled as a Markov Decision Process, and according to a deep double Q network with prioritized experience replay to learn the routing-decision process. Meanwhile, a reward function was designed to obtain a better convergence rate, and the analytic hierarchy process (AHP) was used to analyze the weights of the considered factors in the reward function. Finally, to verify the effectiveness of DSEGR, we conducted simulation experiments to analyze network performance. The simulation results demonstrate that our proposed routing protocol remarkably outperforms others in packet delivery ratio and has a faster convergence rate.

## 1. Introduction

Hardware and communication technologies have developed by leaps and bounds in recent years, making UAVs more agile, more robust and lower cost. The applications of UAVs are becoming more and more extensive, such as wildfire monitoring [1], border surveillance and reconnaissance [2], post-disaster communication aid [3], pesticide spraying for farming [4], and assist sensor network communication [5]. The UAV ad hoc network is more efficient in dealing with complicated multitasking and has higher scalability than a single UAV.

A UAV ad hoc network (UANET) is a particular ad hoc network similar to VANET and MANET. The similarities among them are: each node can move freely and communicates with other nodes; the differences are: the nodes move faster for UANETs and network density is sparse in some scenarios [6]. Furthermore, UAVs can move in 2D and 3D [7] environments with high speed. The longer distances and higher speeds will consume a lot of energy for communication, which is a fatal problem for energy-limited UAVs, and lead to link interruption when nodes’ energy is exhausted. The high-speed mobility of UAVs will bring about frequent topology change and link instability. Moreover, UANET environments are more flexible and changeable. Therefore, considering the challenges mentioned above in UANETs, we must design an adaptive and intelligent routing protocol to select the next hop for better network performance in UANETs.

The primary purpose of routing is that a source node sends packets to a destination node by forwarding through some intermediate nodes. There are three categories concerning routing protocols: traditional routing protocols, heuristic routing protocols and reinforcement-learning (RL)-based routing protocols. Traditional routing protocols mainly include four classes: (1) Proactive protocols, such as optimized link state protocol (OLSR) [8], which is a table-driven method for forwarding packets in a timely manner based on global network topology. However, it needs to frequently update routing tables, resulting in excessive overhead. (2) Reactive routing protocols, like ad hoc on-demand distance vector (AODV) [9], which can reduce overhead, but it takes a long time to re-establish a new route when the network topology changes or route failure occurs. (3) Hybrid protocols, the like zone routing protocol (ZRP) [10]. This combines the advantages of proactive and reactive routing. However, it is challenging to maintain the gathering of information concerning high-dynamic nodes and link behavior. (4) Geographic location protocols, for instance, GPSR [11], which utilize the location information for greedy forwarding of packets and perimeter forwarding when faced with a void area. Although GPSR is superior to non-position-based routing protocols, perimeter forwarding causes large delays which may severely impact network performance. In summary, due to the unique features of UANETs, it is tough to adapt these protocols to highly dynamic UANETs. Many studies have proposed modifications of the traditional routing protocols to adapt to UANETs [12,13]. Although traditional routing protocols are simple and easy to implement, their major drawbacks are a lack of intelligence and autonomy in terms of adapting to high-speed mobility, complex environments, and varied flight tasks in UANETs. Some researchers have been inspired by laws of nature or experience with specific problems, proposing heuristic routing protocols. In [14,15], a genetic algorithm-based routing protocol to improve the efficiency of MANET was proposed. Ant colony optimization has been used for routing protocol [16,17]. However, in all of these protocols, only simple intelligent interactions are implemented, lacking explicit theoretical support, and global optimal solutions cannot be guaranteed.

In order to overcome the above problems, some researchers have proposed reinforcement-learning (RL)-based routing protocols that are intelligent and highly autonomous in UANETs. As we know, RL is an essential part of machine learning, and is inspired by behaviorist psychology; agents can make decisions by interacting with an environment so as to maximize the cumulative reward. Each UAV is regarded as an agent for RL-based routing protocols and learns to select the next hop based on the designed reward function and the algorithmic model to maximize the overall performance of the network according to the different optimization criteria. Currently, most researchers use value-based RL to solve routing problems in UANETs [18,19,20,21]. However, these value-based reinforcement-learning routing protocols mainly operate by establishing Q-Table to select the next hop, which consumes a lot of memory space when the network scale is large. Meanwhile, It needs to retrain the Q-Table when some nodes join or leave, which leads to poor extensibility. Therefore, deep-neural-network-based reinforcement learning is proposed to solve the above routing problems [22]. However, the majority of the aforementioned studies do not comprehensively consider the limited energy problem and the link stability, which seriously affect the network performance.

Motivated by the above considerations, a distributed model-free deep-reinforcement-learning algorithm is proposed to optimize the geographical routing protocol. When a node selects the next hop, it considers location information, link stability, and energy information. Finally, nodes can learn how to choose optimal action quickly to maximize network performance through constant training. The main contributions of this paper can be summarized as follows:

- We introduce a link-stability evaluation indicator, which uses the variance of distance between nodes over a period of time to measure the degree of link stability for decreasing high mobility caused packet loss.
- We use the ARIMA model to predict the neighbor nodes’ residual energy to prevent premature node death, which can achieve energy balance and decrease packet loss.
- A double deep Q network with prioritized experience replay is used to assist in making routing decisions efficiently. We take geographical location, residual energy, and link stability into account when selecting the next hop. According to the above-considered factors, a more appropriate reward function is designed to make the algorithm converge more quickly.
- We conducted extensive experiments and analyzed various performance metrics to verify the advantages of our proposed protocol, The results show that network performance of DSEGR in terms of packet delivery rate and convergence rate is better than the compared routing protocols.

The remainder of this paper is arranged as follows. Section 2 reviews the related works on UANETs and indicates the problems concerning the routing protocols. The system model is presented in Section 3. Section 4 describes our proposed DSEGR protocol in detail. Experimental results and network performance analyses are presented in Section 5. Finally, the conclusions and future work are presented in Section 6.

## 2. Related Work

### 2.1. Conventional Routing Protocol

Traditional routing protocols have developed rapidly in the last few decades. The authors of [23] carried out performance tests and comparisons concerning classical routing protocols for AODV, OLSR, Dynamic Source Routing (DSR), and the geographic routing protocol (GRP). The simulation results indicate that different routing protocols can be used in different UAV communication network scenarios. Moreover, some studies have proposed amending the traditional routing protocols. The authors of [24] introduced a predictive OLSR (P-OLSR) protocol that takes advantage of the GPS information to predict the quality of the links among the nodes. The experiment’s results show that the P-OLSR protocol performs better than the classic OLSR in average goodput. The authors of [25] proposed the mobility- and load-aware OLSR (ML-OLSR) routing protocol. The results of the study show improved delay performance and packet delivery rate compared to OLSR. However, each node consumes large amounts of energy for movement and communication, especially for energy-limited small UANETs. Thus, the ML-OLSR protocol lacks an energy metric to achieve a better performance. The authors of [26] proposed a new mobility model based on spiral line (SLMM) for aerial backbone networks. With the help of the mobility model, it can improve AODV. These routing protocols are all based on topology and have a low degree of autonomy. They cannot achieve a better performance to adapt to the high mobility, the complex flight environment, and the diverse flight tasks. In addition, the routing protocols based on topology aim at building the whole network routing table, which increases the maintenance costs. Thus, location-aided routing (LAR) [27] uses geographic location information to reduce the routing overhead. The later classic greedy peripheral stateless routing (GPSR) protocol [11] is proposed. Every UAV periodically broadcasts geographical location information to assist packet forwarding. GPSR uses greedy forwarding to reduce the end-to-end delay. With the increasing number of network nodes, the GPSR protocol has stronger expansibility, which is more suitable for UANETs. However, it only achieves local optimality and causes excessive routing overhead when nodes are faced with a void area. To overcome the excessive overhead caused by perimeter forwarding. In [28] a game theory was used to select the next-hop node considering the forwarding angle and distance in the perimeter forwarding stage. In reference [29], the authors proposed ECORA, which considers the positioning prediction and link expiration time to provide a better quality of experience (QoE).

### 2.2. Heuristic Routing Protocols

Researchers have come up with some heuristic routing protocols, which have primary intelligence and can appropriately optimize and adjust the network communications temporally. The authors of [30] integrated Tabu-search meta-heuristics and simulated annealing to improve local-search results in discrete optimization for geographical routing protocol in the Delay-Tolerant Network. The results show the Tabu list improves the packet delivery ratio, and if Tabu is used, a better performance can be obtain with the simulated annealing routing strategy. In [30], an ant colony optimization algorithm and the Dynamic Source Routing algorithm are proposed. The pheromone level is calculated by the distance, the congestion level, and the stability of a route. A new pheromone volatilization mechanism is proposed; judging by the results, the proposed algorithm outperforms traditional algorithms in terms of network performance for a battle environment. In [31], the authors extended the Gaussian–Markov mobility model into the fitness function of the bee colony algorithm (BCA) to predict the link expiration time, and then used BCA for the route discovery of FANETs in the 3D environment [32]. A genetic-algorithm (GA)-based ad hoc on-demand multi-path distance-vector routing protocol is proposed [33], which utilizes a fitness function to optimize the path based on energy consumption. The proposed routing protocol extends the network’s lifetime. Although a heuristic algorithm can employ simple intelligence to manage an ad hoc network, it is more suitable for solving static optimization problems. A heuristic routing protocol will relearn when nodes join and leave, and which lack of scalability and consume considerable computation time for high mobility UAV networks.

### 2.3. Reinforcement Learning Based Routing Protocol

With the development of artificial intelligence, RL technologies have shown prominent advantages in dynamic routing optimization. Some works utilize deep RL to autonomously learn routing decisions, which causes nodes to have independent processing capabilities, and full intelligence to handle complex tasks and understand an environment. The paper of Boyan and Littman [34] is a groundbreaking work that used RL in a communication network. After its publication, many studies followed the original RL idea to improve the routing performance according to different features of networks and performance requirements [35,36,37]. The QGrid [38] routing protocol divides the whole network into different grids then selects the next hop with the optimal grid which can avoid void areas and achieve the optimal distance to the destination, but QGrid does not consider link status, which may cause a suboptimal solution. To settle this problem, the authors of [39] proposed a Q-learning-based geographic routing protocol (QGeo), which considers the packet link error and location error to improve the packet delivery rate. However, the limitations of the paper are that its methods present difficulties concerning convergence when Q-table is very large. In [40], the authors proposed a hybrid routing protocol which proposed position prediction to overcome the directional deafness problem in the MAC layer and, furthermore, they proposed a Q-learning routing protocol in a network layer, but it has the same routing problems as QGeo. Therefore, it cannot be applied to large-scale networks. In [41], the authors proposed a full-echo Q-routing algorithm with simulated annealing interference (FEQSAI) to adaptively control the exploration rate of the algorithm. It is an online learning algorithm that learns by adjusting the adaptive temperature parameter which is related to the energy and the speed of nodes. It is suitable for emergency FANET scenarios that cannot be trained in advance, but it cannot obtain a better performance at the beginning and needs to retrain in new scenarios. In order to adapt to large-scale networks, a deep Q network enhanced GPSR protocol (QNGPSR) is proposed in [42]; it improves routing performance by using a deep Q network and two-hop node estimation to avoid the probability of packet forwarding to void areas. However, QNGPSR cannot register load information. To solve this problem, a traffic-aware QNGPSR (TQNGPSR) protocol was proposed in [43]. TQNGPSR achieves load balance and adapts to large traffic scenarios. In Table 1, we list some of the routing protocols, highlighting the characteristics of different protocols and comparing them with our proposed routing protocol. Although the previous works provide relatively effective improvements, there are still some drawbacks that need to be optimized.

## 3. System Model

In this paper, we study a routing problem in such a scenario where UAVs perform search and rescue missions in natural disasters or air crashes. During the search phase, the UAVs move randomly with high speed in a large area to find urgent and critical target areas quickly. In the rescue phase, multiple UAVs collaboratively execute tasks in a relatively small and fixed area; the speed of the UAVs is low and the relative displacements between the nodes are not very large. In order to efficiently complete the search and rescue missions, UAVs need to interact with the other nodes. The communication radius of UAVs is R. The initial energy and the type of UAV are all the same. Each node is equipped with GPS.

### 3.1. Link Stability Model

In order to find targets quickly in the search phase, high mobility is one of the major challenges of the UAV routing protocol, and leads to frequent topology changes or communication link interruptions. For the sake of reducing packet loss due to the rapid movement of nodes, we propose a link-stability evaluation indicator. Each node records its own location information during the last five hello moments and stores the location sequence in the hello packet header. A node *i* receives the location sequence $L_{j}=\left[L_{j}\left(t_{1}\right),L_{j}\left(t_{2}\right),L_{j}\left(t_{3}\right),L_{j}\left(t_{4}\right),L_{j}\left(t_{5}\right)\right]$ from its neighbor node *j*. The distance between node *i* and its neighbor node *j* at time $t_{1}$ can be calculated as follows

(1) $$D_{ij}\left(t_{1}\right)=\sqrt{{\left(L_{i}^{x}\left(t_{1}\right)-L_{j}^{x}\left(t_{1}\right)\right)}^{2}+{\left(L_{i}^{y}\left(t_{1}\right)-L_{j}^{y}\left(t_{1}\right)\right)}^{2}}$$

where $\left(L_{i}^{x}\left(t_{1}\right),L_{i}^{y}\left(t_{1}\right)\right)$, $\left(L_{j}^{x}\left(t_{1}\right),L_{j}^{y}\left(t_{1}\right)\right)$ are the coordinates of the nodes *i* and *j* at time $t_{1}$, respectively. Hence, the distance sequence between *i* and *j* during the last five hello intervals is expressed as $D_{ij}=\left[D_{ij}\left(t_{1}\right),D_{ij}\left(t_{2}\right),D_{ij}\left(t_{3}\right),D_{ij}\left(t_{4}\right),D_{ij}\left(t_{5}\right)\right]$. Then, in accordance with the variance of $D_{ij}$, the link change extent between *i* and *j* over a period of time can be evaluated.Then, we can estimate link stability $LS_{ij}$, which is given by

(2) $$LS_{ij}=D\left(D_{ij}\right)=E\left(D_{ij}{}^{2}\right)-E{\left(D_{ij}\right)}^{2}=\sum_{t}^{t_{5}}\frac{D_{ij}^{2}\left(t_{u}\right)}{5}-{\left(\sum_{t}^{t_{5}}\frac{D_{ij}\left(t_{u}\right)}{5}\right)}^{2},u=\left\{1,2,3,4,5\right\}$$

where $E\left(D_{ij}{}^{2}\right)$ and $E{\left(D_{ij}\right)}^{2}$ are the expected distance and the expected square of the distance, respectively. $D\left(D_{ij}\right)$ is the variance of $D_{ij}$. If the variance is equal to zero, the link between nodes *i* and *j* is strictly stable during the last five hello intervals. The smaller $D_{ij}$ is, the more stable the link is.

### 3.2. ARIMA-Based Residual Energy Prediction Model

In the rescue phase, some nodes may be required to stay in the air for a period of time, or flight speed is relatively slow, which will cause nodes to consume less energy in the marginal area, but consume a lot of energy in the central region, i.e., energy imbalance. Eventually, some central nodes die early, leading to sparse network topology, and further decreasing packet delivery rate.

**Figure 1:**
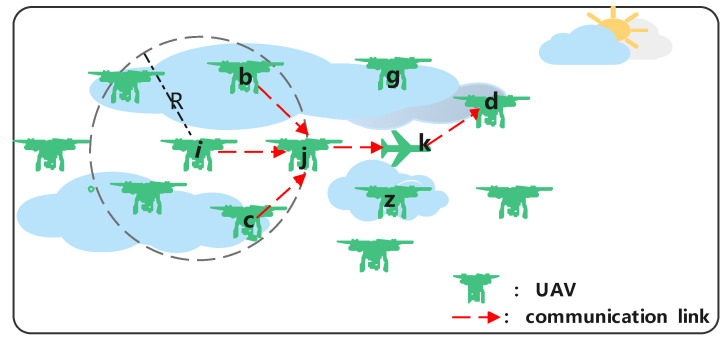
UAV network topology and communication link.

In order to clarify the problem, we use Figure 1 as an example, which shows UAV nodes and communication links in UANETs. Source nodes i,b,c want to send packets to the destination node *d* at different time slots. Then, packets will be forwarded through nodes *j* and *k* successively and finally reach the destination node *d*. Hence, relay nodes *j* and *k* will take on more communication tasks, leading to the nodes becoming congested and more likely to run out of energy. Assuming node *j* is on the verge of death, but node *i* does not receive the latest hello packet from the node, if the node still sends packets to node *j*, it will cause packet loss.

Therefore, we take energy into consideration when selecting the next hop to address the energy imbalance problem. In our scenario, the influence that the external environment, the weather, and some unimportant factors have on energy are ignored. The energy consumption of UAVs is mainly divided into the following three parts: flight energy consumption, send-packet energy consumption, and receive-packet energy consumption. Nodes move at the same velocity magnitude, but in random directions, so the flight energy consumption is basically proportional to the time. The residual energy of node *j* after receiving packets from nodes i,b,c and sending packets to node *k* during a hello interval is denoted as follows.

(3) $$E_{j}^{res}\left(t_{1}\right)=E_{j}^{res}\left(t_{0}\right)-m\cdot P_{size}\cdot \left(E_{tx}+E_{fs}d_{jk}^{2}\right)-n\cdot P_{size}\cdot E_{rx}-E_{fly}$$

where $E_{j}^{res}\left(t_{0}\right)$ is the residual energy of the node at the last hello moment $t_{0}$. $E_{j}^{res}\left(t_{1}\right)$ is the residual energy of the node at the current hello moment $t_{1}$. $E_{fly}$ is the flight energy consumption during a hello interval. $E_{rx}$ and $E_{tx}$ are the energy consumption when transmitting and receiving a packet, respectively. $\varepsilon f_{s}d_{i,j}^{2}$ is the required energy loss in the free-space model; the energy dissipation coefficient of the circuit amplifier is 2.

Meanwhile, each node records its residual energy sequence during the last ten hello intervals and formats the energy sequence in the hello packet header. For example, the residual energy sequence of node *j* is represented as $E_{j}=\left[E_{j}^{\mathrm{res}\;}\left(t_{1}\right),E_{j}^{\mathrm{res}\;}\left(t_{2}\right),\cdots ,E_{j}^{\mathrm{res}\;}\left(t_{10}\right)\right]$. Each node periodically broadcasts its energy sequence to the neighbor nodes by hello packets. For the sake of avoiding nodes dying early due to packet loss, each node uses the ARIMA model to predict neighboring nodes’ residual energy by receiving neighbors’ energy sequences. The ARIMA model is a time-sequence analysis model which can use historical data to predict future values. It has flexible and excellent forecasting performance and lightweight computational cost. Moreover, the reason we used the ARIMA model is that the energy of UAVs is affected by many factors, so the variation pattern of energy consumption is neither stationary nor purely linear or non-linear. Some statistical prediction methods, such as the autoregressive (AR) model, the moving-average (MA) model and the ARMA model, cannot adapt to our scenario. However, the ARIMA model can be applied in a non-stationary time sequence.

Due to UAVs’ residual energy sequence being non-stationary, firstly, we should preprocess the residual energy sequence when a node receives a hello packet from a neighboring node. A d-order differencing is used to transform it into a stationary time sequence, where d is the number of times that the residual energy of the node has been subtracted from past values. Then, we need to check whether the residual energy sequence is a white noise sequence. If it is not, we need to determine the parameters p and q of the ARIMA model by the autocorrelation coefficient (ACF) and the partial autocorrelation coefficient (PACF), respectively. *p* is the order of the auto-regression model, which represents the relationship between the remaining energy of the node at the next moment and the remaining energy of the node in the previous *p* moments. *q* is the order of the moving average model, which describes the relationship between the remaining energy of the nodes at the next moment and the white noise for the previous *q* moments. The Akaike information criterion is used to specify the optimal order (p,q) of the model. After the model order is determined, the mean square error method or the maximum likelihood method is used to obtain the weight coefficients η and φ of the autoregressive-term and the sliding-average-term. According to the ARIMA model established above, node *i* can predict the residual energy of the neighboring node by the following formula

(4) $${\hat{E}}_{j}^{res}\left(t\right)=\sum_{v=1}^{p}\eta_{v}{\hat{E}}_{j}^{res}\left(t-v\right)+w\left(t\right)+\sum_{l=1}^{q}\varphi_{l}w\left(t-l\right)$$

where ${\hat{E}}_{j}^{res}\left(t\right)$ is the predicted residual energy at the next hello moment *t*. ${\hat{E}}_{j}^{res}\left(t-v\right)$ is the residual energy at the previous vth hello moment. w(t) is the Gaussian white noise of residual energy of the node *j* at the next moment t. w(t−l) is the Gaussian white noise at previous lth hello moment. $\eta_{v}$ and $\varphi_{l}$ is the weight coefficients of residual energy and Gaussian white noise at the previous vth and lth hello moments, respectively.

## 4. Proposed Routing Protocol

**Figure 2:**
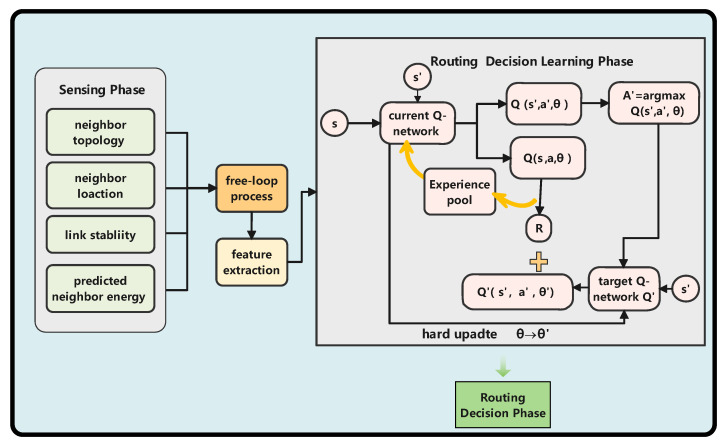
DSEGR protocol architecture.

In this section, we proposed a model-free reinforcement-learning-based geographic routing protocol. The process of routing decisions is simplified as the Markov Decision Process. Our protocol mainly includes the following parts: sensing phase, feature extraction phase, learning phase, and routing decision phase. The routing protocol architecture is shown in Figure 2. The sensing phase is to acquire features. The deep-reinforcement-learning algorithm is used for training nodes in how to make routing decisions. Next, the DSEGR protocol is described in detail.

### 4.1. Sensor Phase

In this sensing phase, each UAV node periodically broadcasts its location by hello packet and maintains its neighbor tables. The neighbor topology table of the node is described as $NT_{i}=\left\{x\in N_{nbr}\left(i\right)|\left(LS_{i,x},E_{x}^{pre},L_{x},NF_{x}\right)\right\}$, where $LS_{i,x},\;$$E_{x}^{pre},$ and $L_{x}$, respectively, represent the link stability between the node *i* and its neighbor node *x* during a period of time, the predicted residual energy of the neighbor node *x* of the node *i* in next hello moment, and the geographical location of a node *x*. $NF_{x}=\left(D_{1d},D_{2d},\ldots ,D_{8d}\right)$ is the topology information of the neighbor node *x*, where $D_{hd}$ is used to denote the farthest distance between the neighbor of node *i* and the destination node *d* in the *h*th region, which is used to estimate the location of the two-hop neighbors of the node in the region, which has already been described in [42].

(5) $$L_{i_{2}}\left(x,h\right)=L_{i}+D_{hd}\left(\cos\frac{(1+2h)\pi }{8},\sin\frac{(1+2h)\pi }{8}\right)$$

In the process of data-packet forwarding, additional information, including the source node *s*, the last-hop node *l*, the destination node *d* and its position information $L_{d}$, and the node set HVN={i,…,l} that has been visited during the packet-forwarding process, are recorded in the data packet. $NF_{i}$ is to be able to evaluate the ability of the two-hop node of node *i* to reach the destination node to avoid forwarding of a packet to a void area. The purpose of recording additional information in a packet is to construct feature values and prevent the formation of loops.

### 4.2. Routing Decision Learning Phase

#### 4.2.1. Markov Decision Process

In this section, the use of deep reinforcement learning (DRL) to learn how to make routing decisions for UANETs is presented. The Markov Decision Process (MDP) is used to simplify the modeling of reinforcement learning. Hence, we should first model the routing problem as an MDP. As we all know, an MDP consists of the following four tuples 〈S,A,P, R〉 where S is a state space, A is an action space, P is the probability of transition from one state to another state, and R is an immediate reward after a state transition. Consider the following scenario: when the node *i* receives a packet, the decision target is to select a neighbor node to forward the packet, while the state is transferred from the current node to the next node, and the process is repeated until the packet delivery fails or succeeds. Based on the above scenario, the MDP can be created as follows.

(1) State space: $S=\left\{s_{1},s_{2},\ldots ,s_{N}\right\}$ where *N* is the total number of nodes in a UANET. the optimal decision of node *i* is decided by the information of its neighbors. Therefore, the features of the node can be extracted as follows

(6) $$s_{i}=\left\{\left(LS_{i,x},E_{x}^{pre},D_{i,d},D_{x,d},D_{i_{2},d},C_{i,x\to x,d},D_{sum}\right)|x\in N_{nbr}\left(i\right)\right\}$$

where $D_{i,d}$ is the distance between node *i* and the destination node *d*; $D_{x,d}$ is the distance between the neighbor node *x* of node *i* and the destination node *d*; $D_{i_{2},d}$ is the minimum distance between the two-hop node $i_{2}$ of nodes *i* and *d*. $C_{ix\to xd}$ is the cosine similarity of the vector from the last hop node *i* to node *x* and the vector from node *x* to the neighboring node $D_{sum}=\sum_{i=1}^{8}\min\left(\left|L_{i}L_{d}\right|-\left|L_{i_{2}}\left(x,h\right)L_{d}\right|,0\right)$ in [37]; the smaller the $D_{sum}$, the greater the probability the two-hop neighbors of the node can reach the destination node and avoid the void area.

(2) Action space: $A=\left\{a_{1},a_{2},\ldots ,a_{N}\right\}$. When the node *i* receives a packet, it needs to select a neighbor as the next hop to forward the packet. Thus, optional action of node *i* is expressed as $a_{i}\in \left\{x|x\in N_{nbr}\left(i\right)\right\}$, where *x* is the the neighbor node *x* of node *i*.

(3) Transition probability: The probability of transition between the node *i* and its neighbor node is determined by the environment, and it is random and unknown.

(4) Reward function: The aim of design reward function R is to make nodes learn how to take a action and achieve faster convergence performance. The reward function of node *i* selecting its neighbor node *x* is described as

(7) $$R_{i,x}=\left\{\begin{array}{cc} 100, & \;\mathrm{if}\;x\;\mathrm{is}\;\mathrm{destenation}\\ -w_{1}D_{x,d}+w_{2}E_{x}^{pre}-w_{3}LS_{i,x}, & \;\mathrm{otherwise}\; \end{array}\right.$$

where $w_{1},w_{2},$ and $w_{3}$ are the weights of the distance between node *x* and the destination *d*, the predicted residual energy of node *x*, and the link stability between node *i* and its neighbor node *x*, respectively. $w_{1}+w_{2}+w_{3}=1$. If the neighbor node of node *i* is the destination, the reward is 100. If not, the reward value is calculated by weighting the factors considered above.

**Figure 3:**
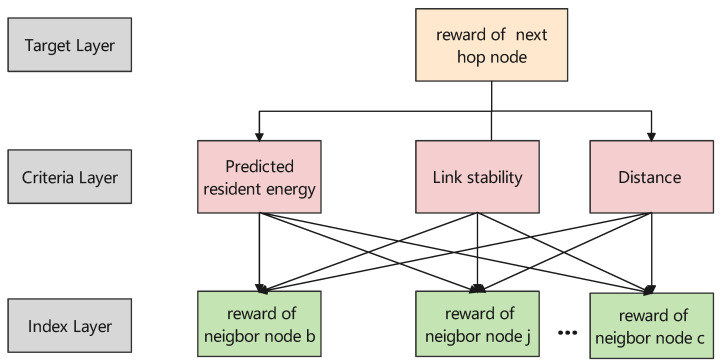
AHP for reward function weight computation.

In order to acquire appropriate weights $w_{1},w_{2},$ and $w_{3}$, The analytic hierarchy process (AHP) method is used. The AHP structure of the reward function is shown in Figure 3. In our scenario, the target layer of the AHP model is the reward of a selected next hop node. The criteria layer is our above-considered factors. The index layer is the reward of different neighbor nodes.

First of all, we need to build the judgment matrix *A*, which is established by comparing each factor of the criteria layer in pairs.

(8) $$A=\left(\begin{array}{ccc} 1 & 2 & 2\\ \frac{1}{2} & 1 & 1\\ \frac{1}{2} & 1 & 1 \end{array}\right)$$

In accordance with the judgment matrix *A*, we can obtain the eigenvector of matrix Aω=(0.5,0.25,0.25), which correspond to the weights of the distance, the predicted energy, and the link stability, respectively. In accordance with Aω=λω, the eigenvalue of matrix *A* is calculated as

(9) $$\lambda_{\max}=\sum_{i=1}^{n}\frac{{\left[Aw\right]}_{i}}{w_{i}}=0$$

Then, we should check the consistency to judge the rationality of the judgment matrix *A*.

(10) $$CR=\frac{CI}{RI}=\frac{\left(\lambda -n)/(n-1\right)}{RI}=0<0.1$$

where *n* is the dimension of the matrix *A*. CR is the consistency ratio, and RI is the random index. If *CR* is less than 0.1, it means the construction of matrix *A* is reasonable and satisfies the consistency condition.

In accordance with the above reward function, as we can see, the weight of the distance is the largest, which means that when the link stability and the neighbors’ predicted energy are the same, the shortest route is a priority, except for a void area situation. When nodes move slowly, the link stability has little effect on the reward function, but the energy factor mainly affects the reward function. When nodes move quickly, link stability is the dominant factor.

#### 4.2.2. DDQN with Prioritized Experience Relay Algorithm

In our scenario, since the action space is discrete, we adapt the value-based RL algorithms. Value-based RL is aimed at learning the optimal policy function $\pi^{*}\left(a_{t}|a_{t}\right)$ based on the action–value function $Q(s_{t}|a_{t})$. The optimal policy function $\pi^{*}$ is denoted as $\pi^{*}\left(a_{t}|a_{t}\right)=P\left(A=a_{t}|S=a_{t}\right)$, and is used to represent the optimal probability of the node selecting the next hop $a_{t}$ in the state $s_{t}$. The action–value function *Q* is expressed as $Q^{\pi }\left(a_{t}|a_{t}\right)=E^{\pi }\left[G|S=s_{t},A=a_{t}\right]$, representing the expected cumulative reward by taking the next hop $a_{t}$ in the state $s_{t}$ with the policy π, where the cumulative reward *G* is denoted as $G=\sum_{t=0}^{T}\gamma^{t}R_{t}$ and represents the sum of the reward values from the current node to the destination node with T hops. γ∈[0,1] is the discount factor, which is used to evaluate the importance of future rewards.

We hope the designed routing protocol can adapt to large-scale UANETs because deep reinforcement learning is a powerful algorithm that combines the advantages of deep learning for perception and reinforcement learning for decision making, which can cause nodes to interact with the environment and intelligently make decisions in UANETs. In [42], the authors use a deep neural network to learn to approximate the action–value function, and there are two *Q* networks; one is the current *Q* network with the parameter set θ, and the other is the target $Q^{\prime }$ network with the parameter set $\theta^{\prime }$. The target *Q*′ network is always greedy in terms of selecting the maximum Q value of the next hop node as the target Q value, which accelerates the network’s convergence, but it leads to overestimation and large biases. In our paper, we use double deep Q-learning (DDQN) [44] to eliminate the problem of overestimation by decoupling the action selection of the target Q value and the calculation of the target Q value. That is to say, the next state *s*′ is input into the current *Q* network to obtain the optimal Q value corresponding to the action *a*′. Then, the action *s*′ and the next state *s*′ are employed to obtain the target Q value in the target Q network. The update of the target Q value is as follows.

(11) $$Q_{\mathrm{target}}=E\left[R_{t+1}+\gamma Q^{\prime }\left(s_{t+1},\underset{a_{t+1}}{\mathrm{argmax}}Q\left(s_{t+1},a_{t+1},\theta_{t}\right);\theta_{t}^{\prime }\right)|\left(s_{t},a_{t}\right)\right]$$

In our work, γ is set as 0.9 based on our experience. The objective function of the *Q* network is to minimize the loss function $L\left(\theta_{t}\right)$, which is shown as:

(12) $$L\left(\theta_{t}\right)=E\left[{\left(Q_{\mathrm{target}\;}-Q\left(s_{t},a_{t};\theta_{t}\right)\right)}^{2}\right]$$

Then, the Adam gradient descent method is used to optimize the loss function. The gradient of the loss function can be depicted as

(13) $$\nabla_{\theta_{t}}L\left(\theta_{t}\right)=E\left[\left(Q_{\mathrm{target}\;}-Q\left(s_{t},a_{t};\theta_{t}\right)\right)\nabla_{\theta_{t}}Q\left(s_{t},a_{t};\theta_{t}\right)\right]$$

In [44], when calculating the target Q value, all samples $e_{t}=\left(s_{t,}a_{t,}r_{t},s_{t+1}\right)$ in the experience replay pool can be sampled with the same probability. However, different samples have different effects on backpropagation. The larger the TD-error, the greater the effect on the backpropagation, where $\;\mathrm{TD}-\mathrm{error}\;=Q_{\mathrm{target}\;}-Q\left(s_{t},a_{t};\theta \right)$. So we adapted the prioritized experience relay in [45], which produces samples where larger absolute values of TD-error are more likely to be produced as samples. Generally, the SumTree method is used to store samples with priority. The convergence rate of the current *Q* network is accelerated with this method.

The parameters of the target $Q^{\prime }$ network can be updated by the newest parameters of the current *Q* network at regular intervals. We use the moving-mean method to update the target network parameter $\theta_{t}^{\prime }=\alpha \theta_{t-1}^{\prime }+\left(1-\alpha \right)\theta_{t}$, which can save storage space and avoid large gaps in the update process that can accelerate learning.

In this paper, the training and run phases adopt two different strategies. In the run phase, we use the ε−greedy method to select the next-hop node, which is depicted as

(14) $$a^{*}=\left\{\begin{array}{cc} \underset{a}{\mathrm{argmax}}Q\left(s,a;\theta \right), & P=1-\epsilon \\ \;\mathrm{others}\;, & P=\epsilon \end{array}\right.$$

where ε is the exploration probability that is used to balance exploitation and exploration in order to avoid falling into a local optimum. Generally, according to our experience, ε is set as 0.05. The 1−ε probability is used to select the next hop with the maximum Q value. The ε probability is used to randomly select a node as the next-hop forwarding node. In addition, we use the softmax function to choose the next hop in the test phase, which can distribute traffic among nodes that have nearly identical Q values. When node *i* needs to forward packets, the softmax policy of node *i* is given by

(15) $$\pi \left(a|s_{i}\right)=\frac{\exp\left(Q\left(a|s_{i}\right)\right.)}{\sum_{a_{i}\in A_{i}}\exp\left(Q\left(a_{i}|s_{i}\right)\right.)}$$

### 4.3. Routing Decision Phase

According to the above-constructed reinforcement learning model, we design the DSEGR protocol. The DSEGR protocol mainly contains neighbor table maintenance and routing decision. Each node maintains and updates its neighbor topology table by the periodic hello packet. Whenever a node receives a packet, the routing algorithm will be executed; a detailed description is shown in algorithm 1. We take node *i* as an instance. First extract features of node *i*, which can be described as $F_{i}=\left\{\left(L_{i},L_{l},L_{d},L_{x},LS_{i,x},E_{x}^{pre},NF_{x}\right)|x\in N_{nbr}\left(i\right)\right\}$, from the neighbor topology table and data packet, where $L_{i},L_{l},L_{d},L_{x}$ is obtained from the data packet. $L_{i,x},E_{x}^{pre},NF_{x}$ is obtained from the NT table information. Then, all neighbor features are input into the RL model to learn how to make an optimal routing decision.
**Algorithm 1 DSEGR routing protocol** **Input:**$L_{i}$: loaction of node *i*; *p*: data packet; $NT_{i}$: topology table of node i; **Output:**
Select next hop with optimal Q value;1:Obtain set of neighbor nodes $A_{i}$ by $NT_{i}$;2:Set of optional neighbor is $A_{i}^{\mathrm{opt}}=A_{i}-HVN$;3:**if**$A_{i}^{\mathrm{opt}}$=*∅***then**4:   $a^{*}=-1$ and packet loss5:**else**6:   **for** *x* in $A_{i}^{\mathrm{opt}}$ **do**7:     Extract $F_{i}^{x}=\left(L_{i},L_{l},L_{d},L_{x},LS_{i,x},E_{x}^{pre},NF_{x}\right)$;8:     Input $F_{i}$ into our RL model and output $Q_{x}$9:     **if** Q network is in training phase **then**10:        Select next hop according to (13);11:        Store $e_{t}=\left(s_{i},a_{i},r_{i},s_{x}\right)$ into experience set $D_{t}=\left\{e_{1},\cdots ,e_{t}\right\}$;12:        Update Q network parameters θ and $\theta^{\prime }$13:     **else**14:        State select next hop using (14)15:     **end if**16:   **end for**17:**end if**

## 5. Simulation Results and Discussion

### 5.1. Simulation Environment and Performance Metrics

In this paper, we use Python 3.6 and the Simpy module to simulate the underlying communication of UANETs. We assume that each UAV is flying at the same altitude. A total of 40∼100 UAVs were randomly distributed in a 2000 m × 2000 m area. Nodes move 10 m/s in the training phase and 1∼20 m/s in the test phase for the DSEGR protocol. The communication radius is 350 m; the hello packet interval is 0.5 s; the DDQN contains four layers corresponding to 5 × 16 × 4 × 1 neurons, and each layer uses SELU as the activation function. Considering that the dimensions of each feature are not the same, the different features are processed by using the Min-Max normalization method, which can speed up the convergence speed of DSEGR. Adam is used as the optimizer. The learning rate α is set to 0.001. The small-batch gradient descent method is used to update parameters, and the batch size is 32. The initial energy of UAVs is 1000 J. The simulation parameter settings of the DSEGR protocol are listed in Table 2.

In this paper, we compare the performance of QNGPSR, DSEGR, FEQSAI, and GPSR protocols in the training and testing phase. To verify the validity of our proposed routing protocol, we analyze the convergence rate of different routing protocols in the training phase, and take the average end-to-end (E2E) delay, packet delivery ratio (PDR), and the time of the first dead node as routing performance evaluation indicators, where average E2E delay can reflect timeliness, PDR demonstrates the reliability of routing algorithms, and the time of the first dead node indicates whether nodes’ energy levels are balanced in a UANET.

### 5.2. Convergence Analysis

**Figure 4:**
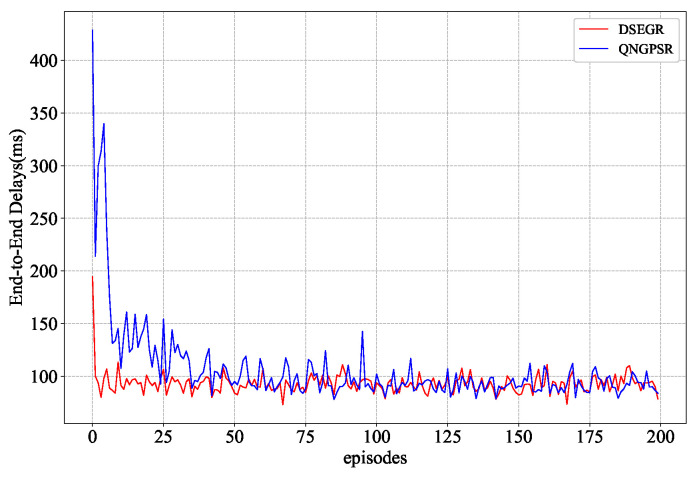
Convergence performance comparison of average E2E delay between QNGPSR and DSEGR in the training phase.

In this section, we mainly analyze the convergence rate of QNGPSR and DSEGR in the training phase since the FEQSAI and GPSR make routing decisions online. One-hundred random maps are used to train the QNGPSR and DSEGR protocol with 200 episodes. Each episode corresponds to 10 s simulation time. Maps are collected from flight trajectories around the Paris Charles de Gaulle Airport in November 2017 from https://www.flightradar24.com/ (accessed on 5 October 2021). The QNGPSR protocol fails to converge due to node movement during the training phase, so QNGPSR nodes are trained in the stationary state. DSEGR is random direction movement. In order to be able to verify the generalization performance of the algorithm, the map used in the testing phase is different from the training phase. The convergence result is shown in Figure 4.

Figure 4 shows the learning curves of QNGPSR and DSEGR. As can be seen, the DSEGR protocol basically converge after five episodes. However, QNGPSR needs 35 episodes to converge. Furthermore, the average E2E delay of DSEGR is close to QNGPSR after the final convergence. Although nodes in QNGPSR are static during the training phase, DSEGR can still obtain a faster convergence speed, which verifies our proposed DDQN with the prioritized experience relay algorithm and designed reward function is reasonable and efficient.

### 5.3. Effects of the Nodes’ Movement Speed

**Figure 5:**
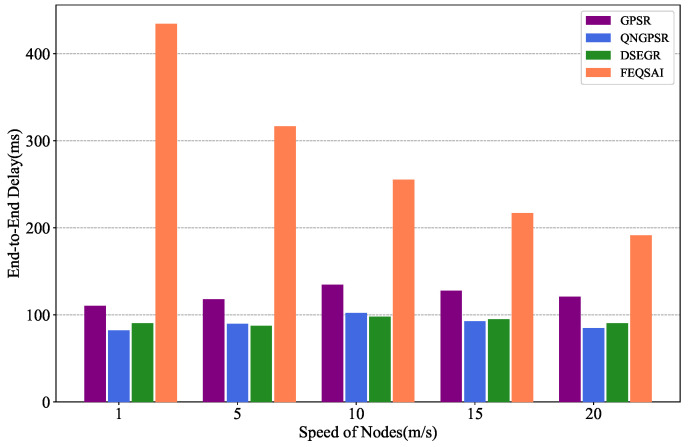
Performance comparison of the end-to-end delay among GPSR, QNGPSR, DSEGR, and FEQSAI with different node movement speeds.

**Figure 6:**
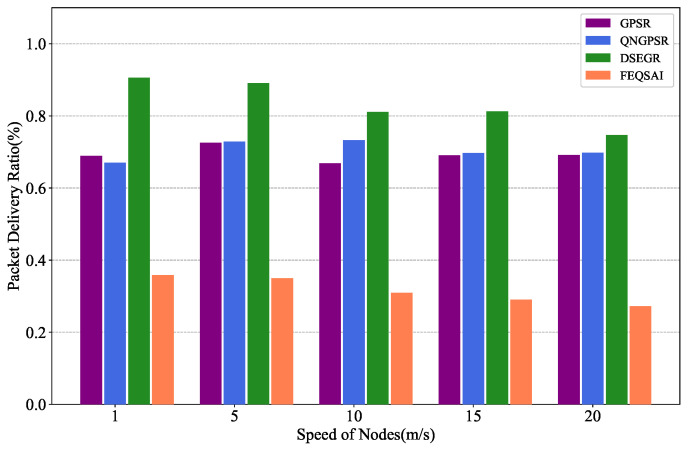
Performance comparison of the PDR among GPSR, QNGPSR, DSEGR, and FEQSAI with different nodes’ movement speeds.

**Figure 7:**
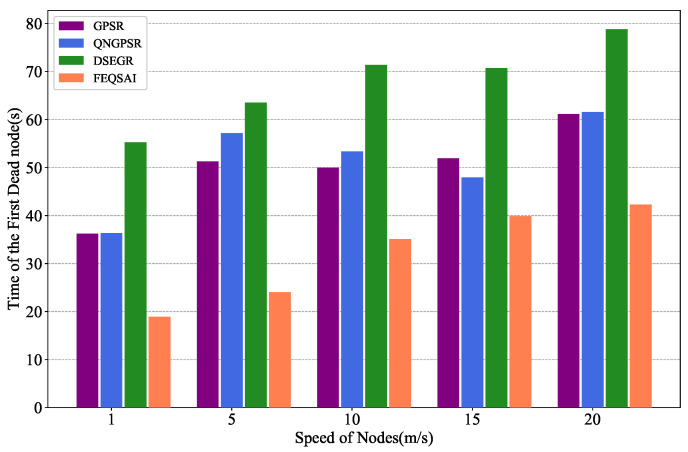
Performance comparison of the time of the first dead node among GPSR, QNGPSR, DSEGR, and FEQSAI with different numbers of nodes’ movement speeds.

In this section, we compare different protocols’ performances with nodes’ movement speed changes between 1 and ∼20 m/s in the test phase. There are 100 nodes in the network. The packet send rate is 1 Hz and the initial energy is 1000 J. The simulation results are shown in Figure 5, Figure 6 and Figure 7.

Figure 5 presents the effects of different nodes’ movement speeds on the average E2E delay. It is obvious that the metric of DSEGR and QNGPSR outperform GPSR and FEQSAI because QNGPSR and DSEGR can reduce the probability of reaching void areas by two-hop neighbor estimation. The average E2E delay of QNGPSR is basically similar to that of DSEGR. As the speed increases, the metric of QNGPSR and GPSR first increases and then decreases; this is because they increase average hop counts. However, once the speed reaches a threshold value, which leads to link-stability decreasing dramatically and packet loss increases, the E2E delay decreases accordingly. The average E2E delay of FEQSAI is the largest since it involves online learning, resulting in the delay being very large at the beginning. It can be seen that, although the maximum average E2E delay difference between DSEGR and QNGPSR is 8 ms at a speed of 1 m/s, the PDR of DSEGR is 26% higher than QNGPSR at a speed of 1 m/s, which is presented in Figure 6. Moreover, the DSEGR has a slight advantage over QNGPSR at 5 m/s and 10 m/s.

Figure 6 shows the effects of different nodes’ movement speeds on the packet delivery ratio. As the speed increases, the PDR of the DSEGR significantly outperforms QNGPSR, GPSR, and FEQSAI. DSEGR considers link stability and predicts the residual of the neighbor node in the reward function, which can greatly reduce the packet loss rate. The main influence factor is mobility when nodes’ speeds are high; thus, unstable links seriously affect the PDR. We can see in the figure that the DSEGR can still achieve higher PDR when the node speed is 20 m/s. At low speeds, the nodes’ residual energy is the main influence factor. However, the DSEGR protocol can achieve a higher PDR. Figure 7 further proves that the time of the first dead node is late for the DSEGR when nodes are at low speeds.

Figure 7 depicts the effects of the different nodes’ movement speeds on the time of the first dead node. As can be seen, the nodes in DSEGR die later than those in QNGPSR, GPSR, and FEQSAI. The reason for this is that DSEGR has an energy prediction model, which can predict the remaining energy of neighbor nodes to balance the energy. Nodes moving at low speeds may lead to certain nodes acting as central nodes. As a result, the central nodes will die early due to node energy exhaustion. With increasing speed, the relative displacement between nodes changes more obviously, and the links become unstable. There are no so-called central nodes; thus, the energy consumption of the nodes will become more balanced, and the time of the first dead node is postponed.

### 5.4. Effects of the Node Density

**Figure 8:**
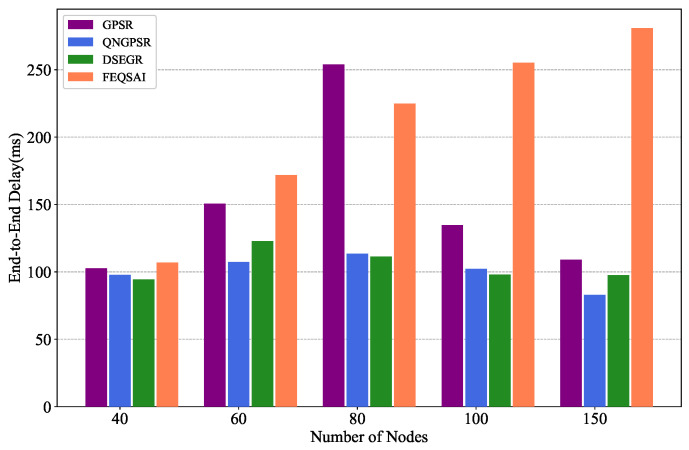
Performance comparison of the average E2E delay among GPSR, QNGPSR, DSEGR, and FEQSAI with different node densities.

**Figure 9:**
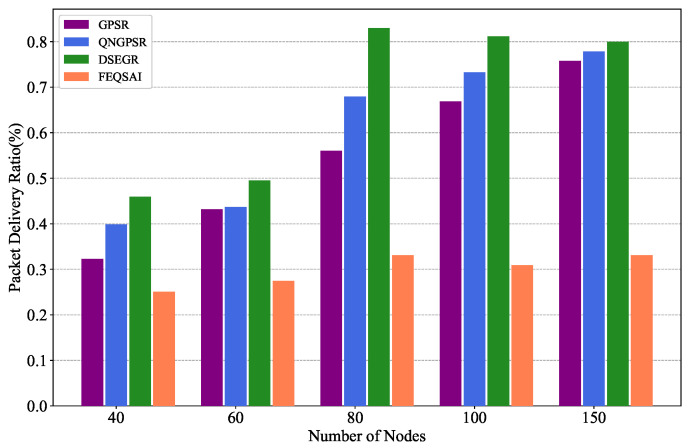
Performance comparison of the PDR among GPSR, QNGPSR, DSEGR, and FEQSAI with different node densities.

**Figure 10:**
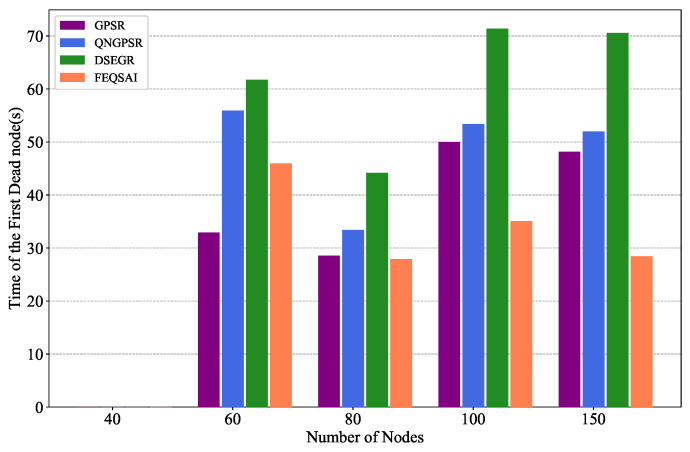
Performance comparison of the time of the first dead node among GPSR, QNGPSR, DSEGR, and FEQSAI with different node densities.

In this section, we compare different protocols’ performances with 40, 60, 80, 100, and 150 nodes in the test phase, separately. The movement speed of nodes is 10 m/s. The packet send rate is 1 Hz and the initial energy is 1000 J. The simulation results are shown in Figure 8, Figure 9 and Figure 10.

Figure 8 shows the effects of the different network densities on the average E2E delay. As can be seen, with the increase in node density, the average E2E delays of DSEGR, QNGPSR, and GPSR increase firstly and then decline. That is because the nodes can achieve more long paths which lead to an initial increase in delays, but the routing void area decreases as the node density increases, which causes an increase in delay. However, FEQSAI needs to relearn online when the number of nodes increases. the average E2E delay of DSEGR increases since Q-Tbale learning complexity increases. So the FEQSAI has the worst delay performance. The average E2E delay of DSEGR is similar to QNGPSR at the same node density. However, GPSR chooses the perimeter forward when it encounters a void area, which causes its average E2E delay to be larger than DSEGR and QNGPSR.

Figure 9 depicts the relationship between PDR and different node densities. It can be seen that, with the total number of nodes increasing in the UANET, each node has more neighbor nodes. Even if some neighbor nodes’ energy is exhausted, there are other neighbor nodes providing assistance to forward packets. Thus, there is an increasing trend in the packet delivery ratio of each protocol. DSEGR significantly outperforms QNGPSR, GPSR, and FEQSAI since DSEGR considers link stability and residual energy prediction. Although FEQSAI considers E2E transmission energy, it is an online learning algorithm and cannot converge in our scenario, so the PDR is the worst.

Figure 10 illustrates the time of the first dead node versus the different node densities. When there are 40 nodes in the network, there are few feasible links. Thus, few packets are effectively and successfully delivered to the destination, which results in network nodes consuming less energy. So there is no dead node. As node density increases, the DSEGR can predict the residual energy of a neighbor node, which can avoid forwarding packets by a low-energy neighor node. Thus, the time of the first dead node is postponed, and the energy consumption of nodes is more balanced. Finally, it can reach a stable state. Hence, the time of the first dead node is almost same when node density is high for DSEGR, GPSR and QNGPSR.

### 5.5. Effects of Packet Send Rate

**Figure 11:**
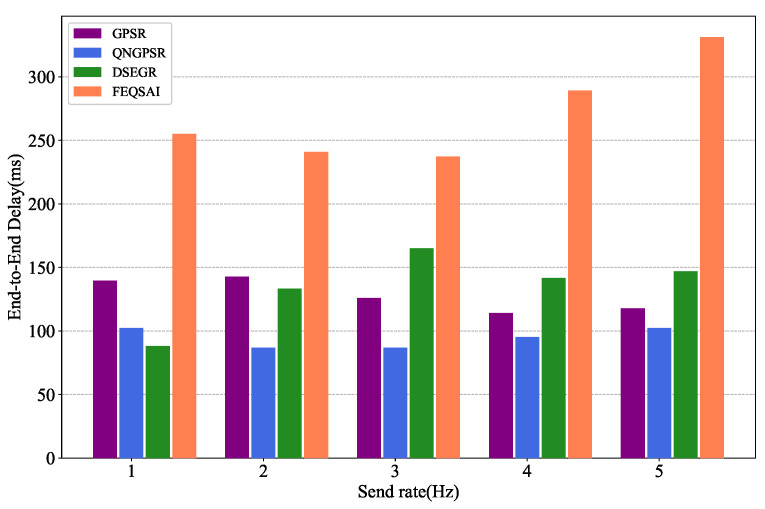
Performance comparison of average E2E delay among GPSR, QNGPSR, DSEGR, and FEQSAI with different packet send rates.

**Figure 12:**
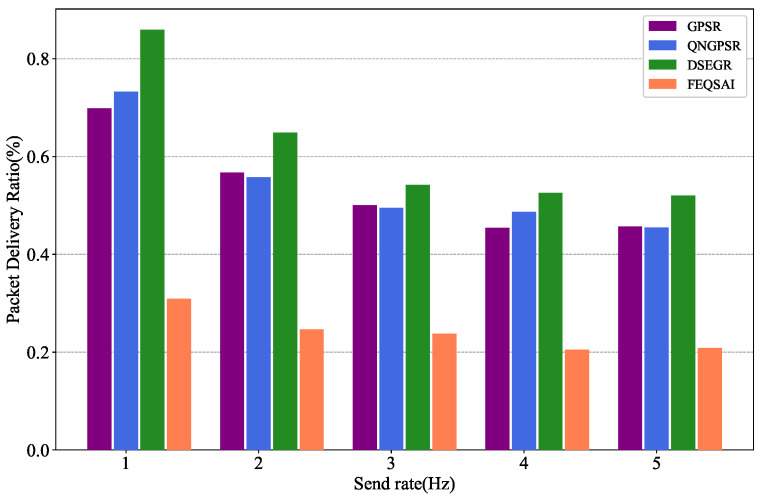
Performance comparison of PDR among GPSR, QNGPSR, DSEGR, and FEQSAI with different packet send rates.

**Figure 13:**
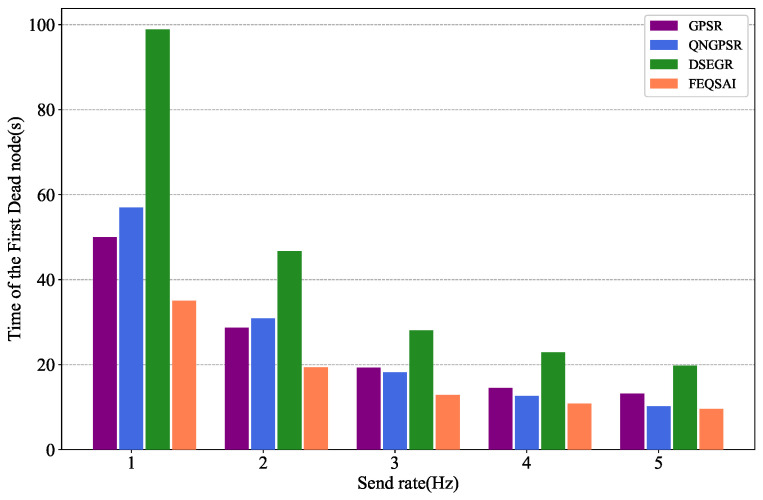
Performance comparison of the time of the first dead node among GPSR, QNGPSR, DSEGR, and FEQSAI with different packet send rates.

In this section, we compare different protocols’ performances with the different packet send rate changes between 1 and 5 Hz in test phase. The node speed is 10 m/s. There are 100 nodes moving in the network and the initial energy is 1000 J. The simulation results are shown in Figure 11, Figure 12 and Figure 13.

Figure 11 presents the effects of the different packet send rates on the average E2E delay. As shown in the figure, when the packet send rate is 1 Hz, DSGER has a lower average E2E delay than GPSR and QNGPSR. As the packet send rate increases, the average E2E delay of DSEGR also increases. The reason for this is that DSEGR adopts an energy prediction model, letting the node die late. Thus, there exist more feasible long paths, which can be verified in Figure 12 and Figure 13.

Figure 12 demonstrates the results of PDR versus different packet send rates. It is clear that with the increase of packet send rate, the PDR of the DSEGR, QNGPSR, and GPSR also decreases. This is because of the nodes’ buffer overflow. DSEGR performs better PDR than the other protocols. The reason for this is that DSEGR selects the next hop considering link stability and the node’s residual energy.

Figure 13 examines the effects of the time of the first dead node on different packet send rates. As we can see, when the send rate is 1 Hz, DSEGR has no dead nodes. Afterward, nodes consume more energy and die early as the packet send rates grows. However, the time of the first dead node for DSEGR is still better than the other protocols. The reason for this is that the DSEGR has an energy predict model, which can balance the energy.

### 5.6. Effects of Initial Energy

**Figure 14:**
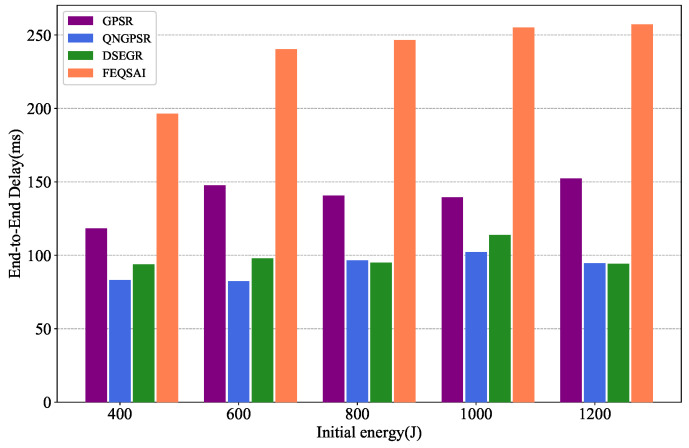
Performance comparison of the time of the first dead node among GPSR, QNGPSR, DSEGR, and FEQSAI with different initial packet energy.

**Figure 15:**
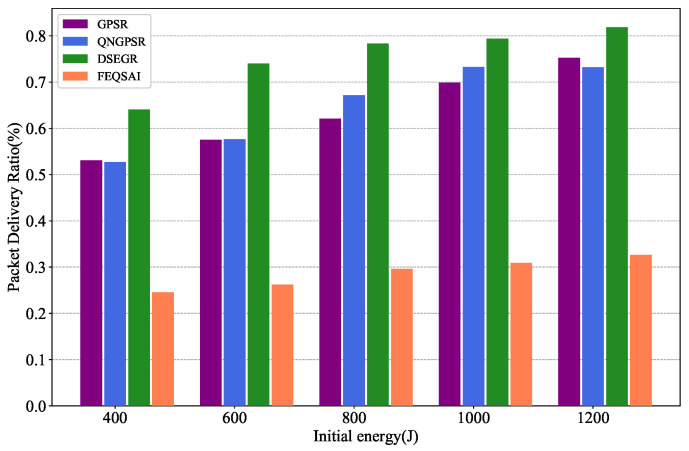
Performance comparison of PDR among GPSR, QNGPSR, DSEGR, and FEQSAI with different initial packet energy.

**Figure 16:**
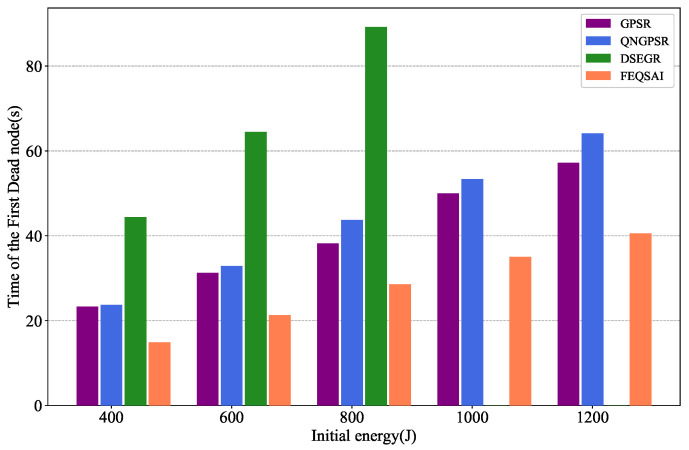
Performance comparison of the time of the first dead node among GPSR, QNGPSR, DSEGR, and FEQSAI with different initial energy.

In this section, we compare different protocols’ performances with the different initial energy changes among 400 and 1200 J in test phase. The speed of nodes is 10 m/s and the packet send rate is 1 Hz. There are 100 nodes moving in the network. The simulation results are shown in Figure 14, Figure 15 and Figure 16.

Figure 14 depicts the relationship between the average E2E delay and different initial energy. As we can see from the figure, as the initial energy increases, the average E2E delay of DSEGR is similar to QNGPSR. The delay of DSEGR and QNGPSR is better than the others since they reduce the routing void area.

Figure 15 illustrates the results of PDR versus different initial energy. With the increase of initial energy, the PDR of all the protocols also increase. This is because the node dies late. It is clear that the DSEGR performs better PDR than the other protocols. The reason for this is that DSEGR can select the next hop considering neighbors’ residual energy. Although FEQSAI considers the E2E transmission energy, which cannot converge due to retraining at different maps, the PDR is lowest.

Figure 16 depicts the effects of the time of the time of the first dead node on different initial energies. As the initial energy increases, the nodes die later and later for all tested protocols. As we can see from the figure, the time of the first dead node for DSEGR is better than the other protocols for different initial energies. There is not even a dead node for larger initial energies, such as 1000 J and 1200 J, because the DSEGR involves an energy prediction method.

## 6. Conclusions

In this paper, we introduced a novel UAV routing protocol to optimize the UAV network performance. Our protocol overcame the shortages of other deep-reinforcement-learning protocols. Meanwhile, we designed a reasonable link stability evaluation indicator to adapt high mobility, and utilized the ARIMA energy prediction model to balance energy. With numerous experiments, simulation results show that DSEGR significantly outperforms GPSR and QNGPSR in terms of PDR. The average E2E delay of DSEGR is close to QNGPSR. The classic GPSR has poor performance metrics. DSEGR can achieve a faster convergence speed. The time of the first dead node is postponed for DSEGR. In summary, our proposed routing algorithm has better robustness and reliability, and is more suitable for performing search and rescue tasks in high dynamic and energy-limited UANETs.

In our work, we only considered a simple scenario. In the future, we will extend the UANET to a more complex integrated ground–air–space scenario. In addition, we will apply multi-agent reinforcement learning in a collaborative manner to obtain better network performance.

## Figures and Tables

**Table 1:** Characteristics of different routing protocols.

Routing Protocol	Energy Management	Link Attribute Perception	Load Balance	Exploration	Quick Adaptation to High-Dynamic Scenarios	Adaptation to Large-Scale Networks	Network Application
GPSR [11]	×	×	×	×	✓	✓	MANET
QGEO [39]	×	✓	×	✓	×	×	Mobile robot networks
QNGPSR [42]	×	×	✓	✓	✓	✓	UANET
TQNGPSR [43]	×	×	✓	✓	✓	✓	UANET
FEQSAI [41]	✓	✓	×	✓	✓	×	FANET
DSEGR	✓	✓	✓	✓	✓	✓	UANET

**Table 2:** Simulation parameter.

Parameter	Value
Movable region	2000 m × 2000 m
Number of UAVs	40∼150
UAV speed	1∼20 m/s
Communication radius	350 m
Initial energy	400∼1200 J
$E_{tx}$	50 nJ/bit
$E_{fs}$	10 pJ/bit/m^2^
$E_{rx}$	50 nJ/bit
Hello interval	0.5 s
Packet size	1 KB
Buffer size	32 KB
Communication radius	350 m
Packet send rate	1 Hz
Packet transmission rate	1 Mbps
Experience relay pool size	2000
Learning rate	0.001
Discount factor	0.9
Packet size	1 KB
Packet send rate	1 Hz
ϵ	0.05

## Data Availability

Not applicable.
